# Associations of financial toxicity with symptoms and unplanned healthcare utilization among cancer patients taking oral chemotherapy at home: a prospective observational study

**DOI:** 10.1186/s12885-023-10580-4

**Published:** 2023-02-10

**Authors:** Yongfeng Chen, Zhenxiang Chen, Haiyun Jin, Yanrong Chen, Jinbing Bai, Guifen Fu

**Affiliations:** 1 Nursing Department, The People’s Hospital of Guangxi Zhuang Autonomous Region, Nanning, China; 2 The Department of Chemotherapy, The People’s Hospital of Guangxi Zhuang Autonomous Region, Nanning, China; 3 Nell Hodgson Woodruff School of Nursing, Emory University, 1520 Clifton Road, 30322 Atlanta, GA USA; 4 Guangxi Academy of Medical Sciences, The People’s Hospital of Guangxi Zhuang Autonomous Region, 530021 Nanning, China

**Keywords:** Oral chemotherapy, Financial toxicity, Symptoms, Unplanned healthcare utilization, Cancer

## Abstract

**Background:**

Cancer patients with financial toxicity experience psychological distress and often miss medical appointments and quit treatments early, which could be a barrier to the effective management of oral chemotherapy drugs at home. This study explores whether financial toxicity predicts symptoms and unplanned healthcare utilization among cancer patients taking oral chemotherapy at home, which will contribute to the safe management of oral chemotherapy.

**Methods:**

Data in this study was from a prospective observational study, which was conducted between October 2018 and December 2019. 151 patients completed the Comprehensive Score for Financial Toxicity at discharge and completed the MD Anderson Symptom Inventory and unplanned healthcare utilization questionnaires after finishing one cycle of oral chemotherapy at home. Regression analyses were conducted to explore the associations of financial toxicity with symptoms and unplanned healthcare utilization.

**Results:**

Among 151participants, 88.08% reported severe or moderate financial toxicity, 43.05% reported symptom interference, and 31.79% reported unplanned healthcare utilization while taking oral chemotherapy at home. Patients between the age of 45-60y (p = 0.042) have higher financial toxicity, while those living in urban areas (p = 0.016) have lower financial toxicity. Patients with worse financial toxicity suffered increased symptoms of fatigue, emotional distress, disturbed sleep, and lack of appetite. Consequently, their mood and personal relation with other significant suffered. However, no statistical differences in unplanned healthcare utilization were found among patients with different levels of financial toxicity.

**Conclusion:**

Middle-aged adults and those living in suburban or rural areas experienced worse financial toxicity than other groups. Patients with worse financial toxicity experienced more severe psychological symptoms (e.g., fatigue, distress, disturbed sleep, and lack of appetite) and affective interference (e.g., mood and relations with others). Identifying at-risk patients is necessary to offer tailored support for psychological symptom management.

## Introduction

Annually, there are about 23.6 million new cancer cases, and 10.0 million patients die from cancer worldwide [1]. Owing to the largest population in the world, more than 4 million new cases and 2.4 million cancer deaths occurred in China in 2016 [2]. Chemotherapy is a cancer treatment that uses one or more anti-cancer drugs to interfere with the fast-growing cancer cells [3]. Nowadays, more oral chemotherapy agents are put into clinical use due to their comparable effectiveness with intravenous chemotherapy and patients’ preference for fewer injections [3]. However, with its required long-term use and less insurance coverage for its costs [4–6], patients with oral chemotherapy suffered more financial toxicities.

Financial toxicity refers to cancer treatment-related objective financial burden and subjective financial distress [7]. It was measured by three dimensions, namely material conditions (e.g., out-of-pocket cost, reduced/lost income, medical debt), psychological response (e.g., distress and concerns due to cost of cancer care), and coping behaviors (e.g., nonadherence of scheduled medication and treatment) [8]. In China, more than 95% of people have basic health-care insurance, among which 25.96% were employee basic medical insurance and 74.04% have urban and rural resident basic medical insurance (including town residents’ medical insurance and new rural cooperative medical insurance) [9]. In general, Chinese patients’ out-of-pocket payments accounted for 28% of total health expenditure in 2020, with Healthy China goals to reduce this to 25% by 2030 [10]. However, compared to chronic pulmonary disease (70.28%), cardiovascular and cerebrovascular diseases (66%), and diabetes (64.29%), patients diagnosed with cancer have the lowest reimbursement ratio (58.33%) [11]. There are regional imbalances in reimbursement ratios as well among the southwestern (51.7%), southern (55.1%), central (57.9%), and eastern (61.5%) areas in China [12]. Furthermore, patients need to pay for cancer treatment-related non-medical expenditures; this accounts for nearly 9% of all medical expenditures, including meals (28.0%), transportation (19.5%), additional nutrition (19.2%), accommodations (15.4%), and hiring personal caregivers (3.6%) [13]. A study showed that out-of-pocket expenditure accounted for nearly 60% of previous-year household income among patients with colorectal cancer [13].

Among the three dimensions used for measuring financial toxicity, financial toxicity reported by material conditions was 6–78%, the psychological response was 61–84%, and coping behaviors were 10–79% among Chinese cancer patients [14]. Further, the psychological financial toxicity (61–84%) [14] in this group of patients is higher than those of patients from publicly funded healthcare countries (7–39%) [15]. Being female, younger age, low or lost income, no health insurance, advanced cancer, chemotherapy, and distance from treatment centers contributed to financial toxicity [7]. Patients with severe financial toxicity are more likely to have a delayed and high-stage diagnosis [16–18], experience worse psychological distress [19], be prone to quit unaffordable treatments [20], miss more medical appointments and medication doses [16–18], and suffer poor treatment outcomes [16–18, 21]. Thus, financial toxicity could be a barrier to the appropriate management of oral chemotherapy drugs at home.

Similar to intravenous chemotherapy, the majority of oral anticancer agents were hazardous drugs [22]. Along with its narrow therapeutic index and safety margin, patients with oral chemotherapy faced severe side effects [23, 24]. A study among cancer patients with oral chemotherapy showed that 79% reported treatment-related toxicities, with 55% being classified as severe [25]. The symptoms patients experienced include nausea, vomiting, fatigue, reduced appetite, disturbed sleep, drowsiness, and memory issues [26]. Without continuous monitoring from healthcare providers, patients with oral chemotherapy need to handle chemotherapy toxicities and cancer-related issues at home by themselves [25, 26]. With limited medical knowledge, these patients had more unplanned utilization of post-discharge services, including emergency room visits, office and outpatient clinic visits, and hospitalization [25–27].

Although financial toxicity is positively associated with cancer patients’ psychological symptoms burden and nonadherence to scheduled treatment, little is known about whether it is related to symptoms and unplanned healthcare utilization among those patients taking oral chemotherapy at home, who experience frequent drug-related adverse effects. Thus, the purposes of this study were (1) to demonstrate the current status of financial toxicity, symptoms, and unplanned healthcare utilization among cancer patients taking oral chemotherapy at home; and (2) to explore whether financial toxicity predicted symptoms and unplanned healthcare utilization among cancer patients taking oral chemotherapy at home.

## Methods

### Design and participants

Data for this study came from a prospective observational study, which was implemented between October 2018 and December 2019 to explore adherence to oral chemotherapy at home [28]. In total, 168 cancer patients taking oral chemotherapy from a provincial level hospital in south China were enrolled in this study. The inclusion criteria included: (1) diagnosed with cancer; (2) received oral chemotherapy and taking 21days as one oral chemotherapy cycle (i.e., the first 14 days take oral chemotherapy drug, and the next 7 days break); (3) no less than 18 years, and (4) were willing to participate. Patients were excluded if they attended other research programs or had a history of diagnosed psychiatric problems.

### Measurements

#### Demographic and clinical data

Demographic data included age, gender, employment status, marital status, educational level, residence, and insurance type. Clinical data included cancer diagnosis, time diagnosed with cancer, tumor stage, Eastern Cooperative Oncology Group score, recurrence, drugs of oral chemotherapy, and times of taking oral chemotherapy at home.

#### Financial toxicity

Financial toxicity was evaluated by the Comprehensive Score for Financial Toxicity (COST) [29]. The COST includes 11 items for calculating the total score. Each item used a 5-point response, with a maximum total score of 44. A higher total score indicates better financial well-being [29]. Based on the total score, financial toxicity was defined by three levels: no/mild (COST ≥ 26), moderate (COST 14–25), and severe (COST 0–13) [30, 31]. The Cronbach’s α coefficient was 0.89 for the Chinese version COST [32].

#### Symptoms

Symptoms were assessed by the MD Anderson Symptom Inventory (MDASI) [33, 34]. The MDASI includes 13 core symptoms and 6 interference items with 11-point responses (0–10). A higher mean score indicates more symptoms severity and interference. Based on the mean score, symptoms severity and interference are defined in 4 levels: no (0), mild (1–3), moderate (4–6), and severe (7–10) [35]. The Cronbach’s α coefficients for the Chinese version MDASI severity and interference items were 0.86 and 0.90 [34].

#### Unplanned healthcare utilization

Unplanned healthcare utilization in this study was defined as using health supports that were not previously planned as part of the schedule of taking oral chemotherapy at home (e.g., routine blood examination, regular follow-up, and going to the hospital for the next cycle of oral chemotherapy). Unplanned healthcare utilization questionnaires in this study were developed from self-reported questions about the utilization of support and health services in Weiss and Lokken’ s study [36]. Patients reported unplanned healthcare utilization while taking oral chemotherapy at home via the following questions in a dichotomous format (yes vs no): calls to doctors for unplanned medical issues, calls to nurses for unplanned medical issues, calls to hospitals for unplanned medical issues, unplanned office/outpatient clinics visits, emergency room visits, and unplanned readmission. Patients answering yes to one of the six items were categorized as unplanned healthcare utilization.

### Ethical consideration

This study was approved by the study hospital and a provincial-level health commission in China (z20180755). Patients who voluntarily participated in this study filled out informed consent forms and could withdraw from the study without impacting their medical services. All the data were kept confidential and only used for research.

### Data collection

After research approval and written informed consent were obtained, the research team asked patients to complete demographic data and COST (for financial toxicity) on the day of discharge. After one cycle of chemotherapy at home (i.e., 21 days from the first day of taking oral chemotherapy drugs), patients completed the MDASI and questionnaires to report symptoms and unplanned healthcare utilization.

### Data analysis

Descriptive statistics were used to describe patients’ demographic and clinical data and the current status of financial toxicity, symptoms severity and interference, and unplanned healthcare utilization. One-Way ANOVA, independent-sample t-test, and ordinal regression analyses were used to explore influencing factors of financial toxicity. One-Way ANOVA, univariate, and multiple ordinal regression were used to explore the relationship between financial toxicity and symptoms. Additionally, univariate and multiple binary regression were conducted to explore the relationship between financial toxicity and unplanned healthcare utilization. The level of significance was set at P < 0.05. All the data were analyzed using SPSS version 23 (IBM Corp., Armonk, NY).

## Results

### Patient characteristics

In this study, 183 eligible patients were contacted, 15 patients responded with no interest or time, and 168 showed a willingness to participate, with an accrual rate of 91.80%. Among the 168 patients recruited, 151 completed the two data collections which were used for data analysis. Table 1 shows the patients’ characteristics. Among the 151 cancer patients taking oral chemotherapy, the ages ranged from 26 to 84, with an average of 54.31. 60.93% of the participants were male, 73.51% lived in an urban area, 97.35% had insurance, 88.74% were diagnosed with gastrointestinal cancer, 87.42% had a tumor stage III or IV, and the time diagnosed with cancer ranged from 1 to 54 months, with a mean time since diagnosis of 9.2 months, and 17.22% suffered cancer recurrence.

**Table 1:** Demographic Data and Its Association with Financial Toxicity among Cancer Patients Taking Oral Chemotherapy at Home

Variables	Frequency N (%)	Financial toxicity		
		Score of COST^1^ M (SD)	F/t	p
Age				
<45y	33 (21.85)	16.85 (7.07)	7.236	0.001**
≥45, <60y	55 (36.43)	15.6 (8.12)		
≥60y	63 (41.72)	20.65 (7.02)		
Gender				
Male	92 (60.93)	18.80 (7.56)	2.691	0.103
Female	59 (39.07)	16.69 (7.95)		
Employment status				
Yes	30 (19.87)	15.73 (6.80)	-1.753	0.082
No	121 (80.13)	18.50 (7.92)		
Marital status				
Married	132 (87.42)	18.51 (7.65)	2.344	***0.020*** ^*^
Single (unmarried, divorced or widowed)	19 (12.58)	14.11 (7.69)		
Educational level				
Primary school or illiteracy	21 (13.91)	18.86 (6.46)	0.979	0.405
Middle	49 (32.45)	16.65 (7.80)		
High school	53 (35.10)	18.00 (8.10)		
College or above	28 (18.54)	19.61 (7.88)		
Residence				
Urban	111 (73.51)	19.10 (7.67)	4.996	***0.008*** ^**^
Suburban	17 (11.26)	16.12 (6.51)		
Rural	23 (15.23)	14.00 (7.66)		
Insurance type				
Medical insurance for staff	65 (43.04)	18.63 (8.61)	2.344	0.075
Medical insurance for town residents	33 (21.86)	20.03 (5.44)		
New rural cooperative medical insurance	49 (32.45)	16.02 (7.73)		
Self-paying	4 (2.65)	14.00 (4.55)		
Diagnosis				
Gastrointestinal cancer	134 (88.74)	18.25 (7.82)	1.487	0.225
Other cancers	17 (11.26)	15.82 (7.06)		
Time diagnosed with cancer				
≤ 6 months	73 (48.34)	19.15 (7.40)	1.834	0.163
>6 months, ≤ 12 months	55 (36.43)	17.30 (7.75)		
>12 months	23 (15.23)	16.00 (8.72)		
Tumor stage				
Stage II	19 (12.58)	16.35 (8.59)	3.949	***0.022*** ^*^
Stage III	65 (43.05)	19.91 (6.88)		
Stage IV	67 (44.37)	16.07 (8.29)		
ECOG score ^2^				
0	21 (13.91)	18.80 (8.22)	2.677	0.072
1	123 (81.46)	18.33 (7.59)		
2	7 (4.63)	11.57 (7.11)		
Recurrence				
Yes	26 (17.22)	15.04 (8.46)	-2.152	***0.033*** ^*^
No	125 (82.78)	18.59 (7.49)		
Drugs of oral chemotherapy				
*Capecitabine*	107 (70.86)	18.47 (7.52)	1.256	0.288
*Tegafur*	40 (26.49)	17.18 (8.23)		
Others	4 (2.65)	13.00 (8.83)		
Times of taking oral chemotherapy drugs at home				
The first time	33 (21.85)	16.42 (7.74)	-1.321	0.188
The second time or above	118 (79.15)	18.44 (7.76)		

N, number; M, mean; SD, standard deviation.

*: p < 0.05, **: p < 0.01, bold: significant result

^1^ COST: the Comprehensive Score for Financial Toxicity, a higher total score indicates better financial well-being.

^2^ ECOG: Eastern Cooperative Oncology Group. ECOG score: 0, fully active, able to carry on all pre-disease performance without restriction; 1, Restricted in physically strenuous activity but ambulatory and able to carry out work of a light or sedentary nature, e.g., light housework, office work; 2, Ambulatory and capable of all selfcare but unable to carry out any work activities; up and about more than 50% of waking hours.

### Financial toxicity and the influencing factors

The total COST score ranged from 0 to 37, with a mean score of 17.98 ± 7.75. Based on the grading level, 18 (11.92%) patients experienced no/mild financial toxicity, 86 (56.95%) patients experienced moderate financial toxicity, and 47 (31.13%) patients experienced severe financial toxicity. Results of One-Way ANOVA and independent-sample t-test (Table 1**)** indicated that age, marital status, residence, tumor stage, and recurrence of cancer were associated with financial toxicity. These significant factors were simultaneously entered into the ordinal regression equation with the level of financial toxicity as the dependent variable (1 = no/mild, 2 = moderate, 3 = severe), and results showed that only age and residence were significant (Table 2). Patients age 45–60 years (p = 0.042) have higher financial toxicity, while patients living in the urban area (p = 0.016) have lower financial toxicity.

**Table 2:** Predictors of Financial Toxicity among Cancer Patients Taking Oral Chemotherapy at Home

Variables	Dependent variable: Financial toxicity				
	Estimate	*SE*	*Wald*	*p-value*	*95% CI*
Model (χ^2^ = 12.078, p = 0.017, R^2^ = 0.091)					
Age (Estimate_≥ 60y_ =0)					
<45y	0.454	0.433	1.096	0.295	-0.396, 1.303
≥45, <60y	0.763	0.375	4.133	***0.042*** *	0.027, 1.499
Residence (Estimate _Rural_ =0)					
Urban	-1.114	0.463	5.781	***0.016*** *	-2.021, -0.206
Suburban	-0.514	0.631	0.663	0.415	-1.751, 0.723

SE, standard error; CI, confidence interval

*: p < 0.05, bold: significant result

### Symptoms and unplanned healthcare utilization at home

Among the 151 cancer patients taking oral chemotherapy at home, 140 (92.72%) patients reported symptoms, and 65 (43.05%) patients reported symptom interference. The most severe symptoms were: numbness/tingling (2.05 ± 2.09), lack of appetite (1.95 ± 2.18), and fatigue (1.86 ± 2.31). The most frequent interference of symptoms in daily life was: general activity (1.16 ± 2.08), working (1.02 ± 1.88), and mood (0.97 ± 1.84). Furthermore, 48 (31.79%) patients reported unplanned healthcare utilization; of these encounters, office/outpatient clinic visits (20.53%), calls to doctors (12.58%), and readmission (10.60%) were the most prevalent.

### Relationship between financial toxicity and symptoms

Results of One-Way ANOVA (Table 3) showed patients with severe financial toxicity reported a higher level of overall symptoms severity and interference at home. Specifically, patients with severe financial toxicity suffered worse pain, fatigue, disturbed sleep, emotional distress, lack of appetite, and drowsiness; additionally, they experienced more symptoms interference with mood, working, relations with others, walking, and enjoyment of life. To further explore the relationships between financial toxicity and symptoms when considering demographic and clinical data, univariate ordinal regression was conducted using each of the above significant symptoms (e.g., symptoms of pain, fatigue, disturbed sleep, emotional distress, lack of appetite, drowsiness; symptoms interference of mood, working, relations with others, walking, and enjoyment of life) as a dependent variable (1 = no, 2 = mild, 3 = moderate/severe) while each of the demographic and clinical variables were treated as the independent variable. Then, significant demographic and clinical variables and financial toxicity were simultaneously put into the ordinal regression with each of the significant symptoms as the dependent variable (1 = no, 2 = mild, 3 = moderate/severe). Symptoms were influenced by financial toxicity, as shown in Table 4, and patients with severe financial toxicity had higher fatigue (p = 0.035) and lack of appetite (p = 0.002) than those with moderate levels; also, they had higher levels of disturbed sleep (p = 0.002, p = 0.045), emotional distress (p = 0.014, p = 0.033), and interference of relations with others (p = 0.020, p = 0.042) than those with moderate and no/mild level financial toxicity, respectively. Additionally, patients with severe financial toxicity had higher mood interference (p = 0.045) than those with no/mild level of financial toxicity.

**Table 3:** Symptoms among Cancer Patients with Different Level of Financial Toxicity While Taking Oral Chemotherapy at Home

Outcome variables Mean (SD)	Overall (n = 151)	Financial Toxicity^3^					
		No/mild (n = 18)		Moderate (n = 86)	Severe (n = 47)	F	p-value
Symptoms severity at home	1.37 (1.31)		0.95 (0.93)	1.18 (1.32)	1.86 (1.31)	5.376	0.006**
Pain	1.17 (1.99)		0.17 (0.38)	1.08 (1.79)	1.70 (2.51)	4.206	***0.017*** *
Fatigue	1.86 (2.31)		1.56 (2.50)	1.51 (2.06)	2.62 (2.53)	3.796	***0.025*** *
Nausea	1.54 (2.02)		1.33 (2.09)	1.41 (1.93)	1.85 (2.17)	0.834	0.436
Disturbed sleep	1.76 (2.31)		1.39 (1.91)	1.34 (2.03)	2.68 (2.69)	5.726	***0.004*** **
Distress (emotional)	1.25 (2.00)		0.61 (1.09)	1.06 (1.93)	1.83 (2.26)	3.406	***0.036*** *
Shortness of breath	0.71 (1.46)		0.28 (0.83)	0.60 (1.37)	1.06 (1.72)	2.443	0.090
Difficulty remembering	1.28 (1.77)		0.78 (1.31)	1.15 (1.80)	1.70 (1.86)	2.317	0.102
Lack of appetite	1.95 (2.18)		1.83 (2.33)	1.48 (1.90)	2.85 (2.37)	6.486	***0.002*** **
Drowsiness	1.23 (1.85)		0.39 (0.85)	0.99 (1.75)	2.00 (2.06)	7.194	***0.001*** **
Dry mouth	1.25 (1.63)		1.11 (1.45)	1.03 (1.54)	1.70 (1.79)	2.682	0.072
Sadness	0.75 (1.51)		0.17 (0.38)	0.78 (1.59)	0.94 (1.61)	1.722	0.182
Vomiting	0.97 (1.73)		0.89 (2.03)	0.79 (1.36)	1.32 (2.16)	1.448	0.238
Numbness/Tingling	2.05 (2.09)		1.83 (1.86)	2.16 (2.16)	1.94 (2.08)	0.289	0.750
Symptom interference at home	0.91 (1.53)		0.21(0.39)	0.76 (1.33)	1.45 (1.94)	5.405	***0.005*** **
General activity	1.16 (2.08)		0.24 (0.66)	1.12 (2.03)	1.57 (2.40)	2.694	0.071
Mood	0.97 (1.84)		0.29 (0.69)	0.81 (1.72)	1.49 (2.22)	3.420	***0.035*** *
Working	1.02 (1.88)		0.29 (0.59)	0.87 (1.75)	1.55 (2.27)	3.524	***0.032*** *
Relations with others	0.66 (1.61)		0.06 (0.24)	0.51 (1.45)	1.15 (2.02)	3.845	***0.024*** *
Walking	0.81 (1.90)		0.12 (0.33)	0.59 (1.60)	1.47 (2.50)	4.726	***0.010*** *
Enjoyment of life	0.87 (1.80)		0.24 (0.44)	0.66 (1.51)	1.47 (2.39)	4.390	***0.014*** *

*: p < 0.05, **: p < 0.01, bold: significant result

^3^ Financial Toxicity: higher total score of the Comprehensive Score for Financial Toxicity (COST) indicates better financial well-being. COST ≥ 26 means no/mild financial toxicity, COST 14–25 means moderate financial toxicity, COST 0–13 means severe financial toxicity.

**Table 4:** Symptoms Influenced by Financial Toxicity Among Cancer Patients Taking Oral Chemotherapy at Home

Variables	*Estimate*	*SE*	*Wald*	*p-value*	*95% CI*
Dependent variable: Fatigue					
Financial Toxicity (Estimate _Severe_=0)					
No/mild	-0.506	0.550	0.845	0.358	-1.584, 0.573
Moderate	-0.746	0.353	4.461	***0.035*** *	-1.437, -0.054
Dependent variable: Disturbed sleep					
Financial Toxicity (Estimate _Severe_=0)					
No/mild	-1.073	*0.536*	*4.003*	***0.045*** *	-2.124, -0.022
Moderate	-1.073	*0.346*	*9.597*	***0.002*** **	-1.752, -0.394
Dependent variable: Distress (emotional)					
Financial Toxicity (Estimate _Severe_=0)					
No/mild	-1.283	0.602	4.537	***0.033*** *	-2.463, -0.102
Moderate	-0.867	0.354	6.012	***0.014*** *	-1.561, -0.174
Dependent variable: Lack of appetite					
ECOG score (Estimate _score 2_ =0)					
0	-2.174	0.908	5.734	***0.017*** *	-3.953, -0.395
1	-1.642	0.805	4.155	***0.042*** *	-3.220, -0.063
Financial Toxicity *(Estimate*_*Severe*_*=0)*					
No/mild	-0.590	0.539	1.196	0.274	-1.647, 0.467
Moderate	-1.135	0.359	9.984	***0.002*** **	-1.840, -0.431
Dependent variable: mood interference					
Age ( Estimate_≥ 60y_ =0 )					
<45y	0.686	0.523	1.718	0.190	-0.340, 1.711
≥45, <60y	1.003	0.462	4.708	***0.030*** *	0.097, 1.909
Financial Toxicity (Estimate _Severe_=0)					
No/mild	-1.659	0.827	4.027	***0.045*** *	-3.280, -0.039
Moderate	-0.644	0.390	2.734	0.098	-1.408, 0.120
Dependent variable: interference of relations with others					
Financial Toxicity (Estimate _Severe_=0)					
No/mild	-2.200	1.080	4.150	***0.042*** *	-4.318, -0.083
Moderate	-0.955	0.411	5.392	***0.020*** *	-1.761, -0.149

SE, standard error; CI, confidence interval

*: p < 0.05, **: p < 0.01, bold: significant result

Level of Financial Toxicity: higher total score of the Comprehensive Score for Financial Toxicity (COST) indicates better financial well-being. COST ≥ 26 means no/mild financial toxicity, COST 14–25 means moderate financial toxicity, COST 0–13 means severe financial toxicity

### Relationship between Financial Toxicity and Unplanned Healthcare utilization

Significant results of univariate and multiple binary regression with unplanned healthcare utilization (1 = no, 2 = yes) as dependent variables are shown in Table 5. The univariate regression analysis indicated that patients with severe financial toxicity (1 = no/mild, 2 = moderate, 3 = severe) experienced more unplanned healthcare utilization than those with moderate (p = 0.037). However, considering significant demographic and clinical variables and symptoms, no difference was found among patients with different levels of financial toxicity. Although, patients with cancer recurrence were 4.96 times more likely to make use of unplanned healthcare utilization while taking oral chemotherapy at home.

**Table 5:** Relationship between Financial Toxicity and Unplanned Healthcare Utilization among Cancer Patients Taking Oral Chemotherapy at Home

Variables	Dependent variable: Unplanned Healthcare Utilization					
	*B*	*SE*	*Wald*	*p-value*	*Exp (B)*	*95% CI*
Univariate binary regression						
Financial toxicity (B _severe =_ 0)						
No/mild	-1.039	0.638	2.650	0.104	0.354	0.101, 1.236
Moderate	-0.794	0.381	4.336	***0.037*** *	0.452	0.214, 0.954
Marital status (B _single_= 0)						
Married	-1.038	0.499	4.329	***0.037*** *	0.354	0.133, 0.942
Recurrence (B _no_ = 0)						
Yes	1.537	0.452	11.550	***0.001*** **	4.650	1.917, 11.282
Pain (B _Moderate/severe_ = 0)						
No	-1.774	0.545	10.590	***0.001*** **	0.170	0.058, 0.494
Mild	-0.663	0.583	1.293	0.255	0.515	0.164, 1.616
Disturbed sleep (B _Moderate/severe_ = 0)						
No	-1.322	0.461	8.221	***0.004*** **	0.267	0.108, 0.658
Mild	-0.341	0.471	0.524	0.469	0.711	0.283, 1.790
Distress (emotional) (B _Moderate/severe_ = 0)						
No	-1.084	0.490	4.896	***0.027*** *	0.338	0.129, 0.884
Mild	-0.539	0.543	0.985	0.321	0.583	0.201, 1.691
Shortness of breath (B _Moderate/severe_ = 0)						
No	-1.172	0.596	3.869	***0.049*** *	0.310	0.096, 0.996
Mild	-0.395	0.687	0.331	0.565	0.673	0.175, 2.588
Lack of appetite (B _Moderate/severe_ = 0)						
No	-1.225	0.469	6.831	***0.009*** **	0.294	0.117, 0.736
Mild	-0.658	0.447	2.172	0.140	0.518	0.216, 1.243
Dry mouth (B _Moderate/severe_ = 0)						
No	-1.127	0.558	4.069	***0.044*** *	0.324	0.108, 0.969
Mild	-0.302	0.555	0.296	0.586	0.739	0.249, 2.194
Multiple binary regression						
Recurrence (B _no_ = 0)						
Yes	1.601	0.538	8.860	***0.003*** **	4.959	1.728, 14.234

SE, standard error; CI, confidence interval

*: p < 0.05, **: p < 0.01, bold: significant result

Symptoms: assessed by the MD Anderson Symptom Inventory. Based on the score, symptoms severity and interference are defined in 3 levels: no (0), mild (1–3), and moderate/ severe (4–10)

## Discussion

This study explored the current status of financial toxicity and its relationship with symptoms and unplanned healthcare utilization among cancer patients while taking oral chemotherapy at home. Results showed that most patients reported moderate or severe financial toxicity. Middle-aged adults and those who lived in rural or suburban areas had worse financial toxicity. Patients with worse financial toxicity suffered severe symptoms of fatigue, distress, disturbed sleep, and lack of appetite; they also suffered severe symptom interference of mood and relations with others. However, no statistical difference was found in unplanned healthcare utilization among cancer patients with different levels of financial toxicity.

In this study, nearly 90% of patients reported moderate or severe financial toxicity, which is higher than Esselen et al.‘s results showing 47% of gynecologic cancer patients from an American medical center experiencing financial toxicity [30], Mejri et al’s study with 80% financial toxicity among cancer patients from a University Hospital in Tunisia [37], and Xu et al.’s systematic review of 61–84% Chinese cancer patients experienced financial toxicity [14]. This discrepancy in results may be explained by the fact that this study included nearly 90% of patients with a cancer stage of III/IV, and these patients had higher health expenditure than those with an early cancer stage [13]. Furthermore, they come from a less developed province in south China, where patients had less insurance reimbursement and more out-of-pocket medical expenditure [12]. In addition, there are more than 80% of patients with high school or lower education backgrounds in this study, and this resulted in a lower overall income for this group [21, 38].

Young and Middle-aged patients with cancer reported higher financial toxicity than old age patients, while middle-aged patients showed significant differences. These differences may be attributed to those young and middle-aged patients being more likely to have dependent children at home and at a higher risk of losing job-related incomes [39], whereas middle-aged adults to be more sensitive to the relationship between financial toxicity and distress [40]. Additionally, older adult experience less job interruption and fewer costs related to raising children [41]. In this study, patients living in rural and suburban areas reported more financial toxicity than those living in urban areas. This may be owing to their lower income, lower insurance reimbursement ratio, higher expenditure on travel costs, and higher incidence of late-stage cancer diagnosis [21, 38]. More considerations, such as the use of remote medical technologies and services, are needed to improve healthcare convenience and reduce the non-medical cost for patients living in rural and suburban areas.

Patients with severe financial toxicity reported severe psychological symptoms of fatigue, disturbed sleep, distress, lack of appetite, and increased interference with their mood and relations with others. These results supported previous results of the positive correlation between financial toxicity and psychological symptoms [19], although they did not offer further evidence to support the links between financial toxicity and physical symptoms. The results offer support for the non-difference status in unplanned healthcare utilization among different levels of financial toxicity in this study. Although patients with severe financial toxicity suffered worse symptoms (e.g., fatigue, disturbed sleep, emotional distress, lack of appetite), these are not the most common reasons (e.g., diarrhea, nausea/vomiting, fever, and skin reaction) for patients to seek additional healthcare supports while taking oral chemotherapy at home [42]. Furthermore, most symptom severity was at a low level, and patients with severe financial toxicity may tolerate symptoms interference to cope with financial toxicity [30, 43, 44]. This study also showed patients with cancer recurrence reported more use of unplanned healthcare resources; however, only 26 (17.22%) patients with cancer recurrence were included in this study, sub-group analyses to identify their specific healthcare needs were not conducted, and further studies to clarify their needs are suggested. Further research is needed to understand the relationship between financial toxicity and unplanned healthcare utilization among cancer patients taking oral chemotherapy at home in longitudinal oral chemotherapy cycles.

Some limitations need to be considered when analyzing this study. First, data in this study came from a prospective study exploring adherence to taking oral chemotherapy at home, and different outcomes were measured with the same sample size, which may overestimate the results’ efficacy. Second, the relationship between financial toxicity and symptoms and unplanned healthcare utilization was only explored in one cycle of oral chemotherapy at home. Although the average time of cancer diagnosis was 9.2 months in this study, it is necessary to study longitudinal patterns of the oral chemotherapy cycle to expand the knowledge base. Third, this study described the current status of unplanned healthcare utilization, though it did not clarify the reasons for the unplanned healthcare utilization. Qualitative studies may be needed to understand the relationship between financial toxicity and unplanned healthcare utilization.

## Conclusion

Cancer patients taking oral chemotherapy suffered severe financial toxicity, especially among middle-aged adults and those living in suburban and rural areas. Patients with worse financial toxicity endured higher symptoms of fatigue, disturbed sleep, distress, and lack of appetite; they also experienced more interference with their mood and relations with others. Nurses and health care providers can use reliable and valid measures, such as COST, to routinely identify those patients with severe financial toxicity to offer tailored support for psychological symptoms management. Although there is a trend of more unplanned healthcare utilization among cancer patients with severe financial toxicity, no statistical difference was found when considering demographic and clinical variables. Further studies to confirm the relationship between financial toxicity and unplanned healthcare utilization are needed.

## Data Availability

The datasets generated and/or analyzed during the current study are not publicly available due to privacy/ethical restrictions but are available from the corresponding author upon reasonable request.
